# A comparative study of hospitalization costs of TKA inpatients before and after National Volume-based Procurement in Guangdong, China: an interrupted time-series analysis

**DOI:** 10.3389/fpubh.2024.1468606

**Published:** 2025-01-09

**Authors:** Anqi Li, Gongduan Li, Dong Han, Manru Fu, Jinghui Chang

**Affiliations:** ^1^School of Health Management, Southern Medical University, Guangzhou, China; ^2^Faculty of Biology, Medicine and Health, The University of Manchester, Manchester, United Kingdom; ^3^The Third Affiliated Hospital of Southern Medical University, Guangzhou, China; ^4^Department of Global Health and Population, Harvard Chan School of Public Health, Harvard University, Boston, MA, United States

**Keywords:** total knee arthroplasty, National Volume-Based Procurement, interrupt time series analysis, cost comparison, healthcare policy

## Abstract

**Background:**

This study aims to explore the impact of the National Volume-based Procurement Policy in Guangdong Province on hospitalization costs for total knee arthroplasty inpatients.

**Methods:**

Interrupted time-series analysis were used to examine the expenses associated with total knee arthroplasty for inpatients at a hospital in Guangzhou between May 10, 2021, and December 26, 2023. The period was divided into two phases based on the implementation of the policy, the pre-policy phase (May 10, 2021, to April 30, 2022) and the post-policy phase (May 1, 2022, to December 26, 2023). A total of ten indicators, derived from patients’ medical records, were analyzed. Indicators included total expenses, self-financed expenses, consumables costs, miscellaneous service fees, diagnostic fees, treatment fees, rehabilitation fees, total Chinese medicine costs, western medicine costs, and the costs of blood and blood products.

**Results:**

A total of 1,196 valid cases were included, with 290 cases before policy implementation and 906 cases after implementation. When the implementation of procurement occurred (May 1, 2022), total expenses ( $\beta_{2}=$ − 28240.17, *p* < 0.001), consumables costs ( $\beta_{2}=$ − 31302.72, *p* < 0.001), and self-financed expenses ( $\beta_{2}=$ − 13674.56, *p*<0.001) showed an instantaneous decreasing trend. The mean total expenses decreased from 65,324.73 CNY per case to 34,465.57 CNY per case, representing a reduction of 30,859.16 CNY per case. Miscellaneous service fees ( $\beta_{2}$ = 440.45, *p* < 0.05), diagnostic fees ( $\beta_{2}$ =746.00, *p* < 0.05), rehabilitation fees ( $\beta_{2}$ =207.36, *p* < 0.001) exhibited an instantaneous and slight increasing trend. After implementation (from May 1, 2022 to December 26, 2023), the total expenses ( $\beta_{3}=-$ 106.95, *p*<0.05), consumables costs ( $\beta_{3}=$ − 65.05, *p*<0.05), diagnostic fees ( $\beta_{3}=$ − 22.44, *p* < 0.05), treatment fees ( $\beta_{3}=$ − 28.01, *p* < 0.05), total Chinese medicine costs ( $\beta_{3}=$ − 9.98, *p* < 0.05), and blood and blood products costs ( $\beta_{3}=$ − 5.88, *p*<0.05) continued to decrease.

**Conclusion:**

The volume-based procurement policy in Guangdong, China has shown initial effectiveness. After it was implemented, total expenses, consumables costs, and self-financed expenses decreased. The structure of hospitalization costs also reflects the slight improvement in healthcare providers’ value and treatment planning decisions. Further attention should be given to the healthcare professionals’ labor value. The empirical analysis results provide a reference for the government further to improve the volume-based procurement for artificial joints.

## Introduction

According to the seventh national population census of China in 2020, individuals aged 60 and above were 190.64 million, accounting for 13.5% of the total population, which increased by 5.44% compared to the sixth census (1). As the aging population continues to grow, the prevalence of obesity rises in the population. The incidence of arthritis-related diseases is steadily increasing.

Total knee arthroplasty (TKA) has become a widely adopted treatment for knee joint diseases, significantly transforming the management of patients with end-stage arthritis following the successful introduction of artificial joints (2–4) In China, approximately 1 to 1.5 million individuals require knee joint replacement surgery (5). However, the high cost of the procedure often deters many patients from undergoing the surgery. The expense of TKA materials, particularly, has led some patients to forgo the surgery (6). Research indicates that consumables represent the largest proportion of hospitalization costs for TKA patients (6). As such, reducing the cost of artificial joint consumables is crucial to lowering the overall cost of TKA.

High-value medical consumables are procured through various models in developed countries, including direct hospital procurement, national or group centralized procurement, and group purchasing organization (GPO) procurement (7). Countries with social health insurance or national healthcare systems, such as the UK, France, Germany, and Australia, commonly employ national centralized procurement (8). The GPO incorporates third-party commercial procurement companies. GPOs play an important part in hospital purchasing in the United States (9). The study indicated that China could improve its centralized procurement system by adopting value-based purchasing and partial evaluation approaches; establishing a standardized coding system; and introducing GPO while optimizing market surveillance mechanisms drawing from developed countries’ experiences (7).

Volume-based Procurement works by buying in bulk to help the hospital negotiate lower prices from manufacturers (10). It refers to a situation in which the quantity of pharmaceuticals or medical consumables to be purchased is communicated in advance to the suppliers. Suppliers then provide quotations based on the required purchase quantity through open bidding and inquiry processes. Essentially, it resembles the concept of collective purchasing, where hospitals act as the units for group purchases. In 2018, the Fifth Plenary Session of the Central Committee for Deepening Reform of the Communist Party of China approved the “Reform Plan for Deepening the Government Procurement System.” It outlined the guiding ideology, principles, and objectives of the National Volume-based Procurement (NVBP) reform aka China’s Volume-based Policy (CVBP) (11). The meeting highlighted the following issues. For the procurement agencies, there is a need to establish a competitive mechanism. For the government, the importance of improving evaluation mechanisms and developing efficient transaction mechanisms was stressed. In terms of management, the meeting stressed the need for a sound supervision system, a modern procurement system with clear responsibilities efficient transaction rules, and advanced technical support. The National Healthcare Security Administration (NHSA), a newly formed agency in 2018, administers most of the NVBP program. Since its initiation, China has organized multiple rounds of centralized volume-based procurement for pharmaceuticals. Over time, the scope of procurement has expanded from chemical pharmaceuticals to biopharmaceuticals and medical consumables. It covers medicines and consumables in areas such as hypertension, diabetes, coronary heart disease, gastrointestinal disease, malignant tumors, and orthopedic trauma.

In June 2021, the NHSA issued guidelines on the organized procurement of medical consumables nationally. The guidelines focus on national organization, alliance procurement, and platform operation. The main objective is to reduce the price of medical consumables, and the burden on patients and ensure better access to medical care (12). In 2022, a policy named “Opinions on Supporting Measures for National Organized Volume-based Procurement and Use of High-Value Medical Consumables (Artificial Joints)” was issued by the NHSA and China’s National Health Commission. The document emphasizes a series of measures, including strengthening policy coordination and leveraging the importance of medical insurance payment and incentives for medical institutions. Measures are aimed at ensuring the successful implementation of NVBP and promoting the high-quality development of the pharmaceutical industry (13).

In 2022, Guangdong Province implemented a volume-based procurement system and issued supporting measures for high-value medical consumables, specifically artificial joints. Relevant issues have been clarified, such as payment standards for medical insurance, defining price adjustments for medical services, and assigning responsibilities to concerned parties (14). Since April 15th, the selected artificial joints have been implemented in 330 hospitals in Guangdong Province, including both public and private hospitals. These efforts aim to improve access to medical services for the insured and effectively reduce the burden of medical consumables for the public.

There is a pressing need to reduce the financial burden of joint replacement surgery in China. Even in more developed regions, the cost of TKA remains high relative to average income levels, necessitating measures to make it more affordable for patients (15). Studies have revealed that NVBP on drugs has promoted pharmaceutical affordability in China (16–18). However, further discussion should be addressed on high-value medical consumables because of their negative correlation with medication costs and their relatively late implementation in Chinese public hospitals (19). Native Chinese studies have found significant price reductions in consumables for coronary vascular interventions (20, 21) under volume-based procurement. Since the implementation of NVBP in 2018, the significant reduction in consumable prices has alleviated the burden on patients and facilitated the promotion of artificial joints (22). However, a study also shows that the total cost of TKA has not decreased, with costs of diagnosis and treatment, anesthesia, nursing, and operation increasing significantly, even though the proportion of material costs is significantly reduced (23). Further analysis is needed considering multiple factors, including the healthcare insurance system and cost structures. The indirect effects of the NVBP policy on other hospitalization costs require ongoing monitoring and investigation. It is crucial to verify whether the reduction in consumable costs has translated into actual savings for patients. The ultimate goal is to ensure that the policy genuinely improves patients’ access to medical services.

Guangzhou, a major metropolitan hub in southern China, is well-known for its advanced healthcare resources. The city’s Healthcare Security Administration has led the way in pioneering innovative procurement strategies, particularly through a market-oriented, group volume-based procurement system. As part of this initiative, Guangzhou has implemented a regional procurement program for medical consumables. This study aims to evaluate the impact of Guangdong Province’s volume-based procurement policy for artificial joints by analyzing hospitalization costs for TKA patients. By comparing costs before and after the policy’s implementation, including changes in specific expense categories, the study seeks to provide practical evidence regarding the affordability of TKA inpatients and offer recommendations for policy refinement.

## Methods and materials

### Data source

Data were extracted from the “Statistical Medical Records Database” of a tertiary hospital in Guangzhou, Guangdong Province, China, spanning from May 10, 2021, to December 26, 2023. Founded in 1953, the hospital is located in one of southern China’s cities renowned for its rich medical resources. The orthopedic department is considered one of the hospital’s leading specialties. In recent years, the hospital’s orthopedic department has been ranked on the prestigious China Science and Technology Evaluation Metrics (STEM) hospital ranking list. The study population comprised valid 1,196 samples who underwent TKA, identified using the Chinese surgical code 81.5400 (total knee arthroplasty).

The following data were collected for analysis: (1) Demographic characteristics of TKA patients: gender, age, type of medical insurance; (2) Clinical information: diagnosis code, admission date, discharge date; (3) Expenses: total expenses, self-financed expenses, consumables costs, miscellaneous service fees, diagnostic fees, treatment fees, rehabilitation fees, total Chinese medicine costs, western medicine costs, blood and blood products costs.

### Patients

To ensure the integrity and accuracy of the included case. Data preprocessing was conducted before the analysis in this study. The data preprocessing criteria were as follows: (1) Exclusion of duplicate data. Sample data with repetitive records were excluded based on demographic characteristics. (2) Removal of missing data. Missing information on variables was excluded. (3) Elimination of abnormal data. Sample case data with obvious logical errors were excluded, such as cases with less than 1 day of hospitalization or cases where the total hospitalization costs did not match the sum of individual costs.

Procurement of artificial joints will be implemented from April 30, 2022, according to the local policy of the Health Security Administration of Guangdong Province (24). Thus, in this study site, the policy intervention date was set for May 1, 2022. The cost analysis was split into 2 periods based on the discharge date of TKA inpatients, as hospitalization expenses are calculated and billed on discharge day. One is a pre-policy implementation period (May 10, 2021, to April 30, 2022). The other is a post-policy implementation period (from May 1, 2022, to December 26, 2023).

### Outcome measures

Ten variables extracted from inpatient medical records were used as outcome indicators to assess the hospitalization expenses of TKA patients. These variables included total expenses, self-financed expenses, consumables costs, miscellaneous service fees, diagnostic fees, treatment fees, rehabilitation fees, total Chinese medicine costs, western medicine costs, and blood and blood products costs. All values were expressed in the Chinese Yuan (CNY).

### Statistical analysis

#### Descriptive statistical analysis

SPSS 26.0 was employed for statistical analysis at this stage. A descriptive analysis was conducted on the demographic characteristics of TKA inpatients before and after policy implementation. The analysis described the sample size (n) and composition (%) in terms of variables such as gender, age, days of hospitalization, and medical insurance type.

Categorical variables were compared using the chi-square test. However, the total hospitalization expenses and individual cost variables in this study were found to deviate from a normal distribution based on the Kolmogorov–Smirnov test. Therefore, a non-parametric Mann–Whitney U rank-sum test was employed to compare the data before and after policy implementation. Multiple linear regression analysis was used to examine the relationship between days of hospitalization and demographic variables. A significance level of *p* < 0.05 was considered statistically significant.

#### Grey relation analysis

Excel 2019 was used to perform grey relation analysis on the data. Grey system theory is a theoretical framework proposed to address the issues of data relationship ambiguity, uncertainty, and randomness in dynamic changes (25). Grey relation analysis, a key element of grey system theory, assesses the level of correlation between a particular indicator and other factors. It determines the relationship between the indicator and these factors by comparing the similarity of the curve shapes. Greater similarity indicates a stronger correlation, while less similarity indicates a weaker one (26). Grey relation analysis was used to rank the influencing components of total expense among TKA inpatients.

#### Interrupted time-series analysis

Stata 16.0 was used for interrupted time-series (ITS) analysis. ITS analysis is a technique utilized to assess data presented in a time-series structure that involves interruptions or interventions, such as the implementation of a policy. This method finds extensive application in public health and policy evaluation domains, with the single-group ITS approach being the predominant methodology. In this research, a segmented linear regression model was utilized for analysis, as outlined in detail in Appendix Table 1. The evaluation of the policy concentrated on investigating both its immediate and enduring impacts. A significance level of *p* < 0.05 was considered statistically significant.

## Results

### General information of TKA inpatients before and after NVBP

After data preprocessing, a total of 1,196 valid cases were included in this study, with 290 cases before policy implementation and 906 cases after implementation. The general information on TKA inpatients is detailed in Table 1.

**Table 1:** General information on TKA in patients before and after NVBP (*n* = 1,196).

Variables	Before NVBP (*n* = 290) [*n* (%)]	After NVBP (*n* = 906) [*n* (%)]	*χ*2	*p*
Gender			0.001	0.973
Male	50 (17.2%)	157 (17.3%)		
Female	240 (82.8%)	749 (82.7%)		
Age Group			46.02	0.43
<40	1 (0.3%)	0 (0%)		
40 ~ 59	38 (13.1%)	107 (11.8%)		
60 ~ 80	232 (80%)	751 (82.9%)		
>80	19(6.6%)	48(5.3%)		
Days of hospitalization			42.14	0.05
<10 days	134 (46.2%)	370 (40.8%)		
10 ~ 20 days	144 (49.7%)	496 (54.7%)		
>20 days	12 (4.1%)	40 (4.4%)		
Medical insurance			7.15	0.21
Resident basic medical insurance	25 (8.6%)	61 (6.7%)		
Employee basic medical insurance	239 (82.4%)	778 (85.9%)		
Fully publicly-funded insurance	4 (1.4%)	20 (2.2%)		
Fully self-pay	19 (6.6%)	44 (4.9%)		
New rural cooperative medical insurance	0 (0%)	1 (0.1%)		
Other insurance	3 (1%)	2 (0.2%)		

^*^p-value<0.05.

In terms of gender distribution, the proportion of females before and after policy implementation was 82.8 and 82.7%, respectively, which was higher than the proportion of males at 17.2 and 17.3%, respectively. TKA inpatients ranged in age from 36 to 89 years, with a mean age of 67.8 ± 7.452 years. The highest proportion of TKA inpatients were aged 60–80 years old, 80% before and 82.9% after policy implementation.

The differences in gender, age group, and type of medical insurance among TKA in patients before and after policy implementation were not statistically significant (*p* > 0.05). However, there was a statistically significant difference in days of hospitalization (*p* = 0.05). Before the policy, an equivalent number of cases had hospital stays of less than 10 days and 10 to 20 days, both less than 50%. After the policy, however, the proportion of TKA in patients with a hospital stay of 10 to 20 days increased to 54.7%. Multiple linear regression indicated that insurance type significantly influenced days of hospitalization. Patients covered by resident basic medical insurance (*β* = −1.060, *p* = 0.038) had shorter hospitalizations, while self-pay (*β* = 1.504, *p* = 0.010) patients experienced longer stays (Appendix Table 2).

### Total and itemized hospitalization expenses of TKA inpatients

#### Total and itemized hospitalization expenses and composition of TKA inpatients before and after NVBP

The results of the Mann–Whitney U test are presented in Table 2, indicating that most variables showed statistically significant differences before and after the implementation of the NVBP policy (*p*-values <0.05).

**Table 2:** Total and itemized hospita lization expenses of TKA inpatients before and after NVBP (*n* = 1,196).

Variables (CNY ^a^/n)	Before NVBP		After NVBP		*Z*	*p*
	x̄±s	M (P25, P75)	x̄±s	M (P25, P75)		
Total expense	65324.73 ± 8860.74	63021.85 (59920.77, 68083.27)	34465.57 ± 8389.86	33300.71 (30062.78, 36626.22)	−25.15	<0.001*
Self-financed expense	32010.8 ± 15239.03	32724.29 (18533.88, 40299.15)	16845.7 ± 8909.48	16864.9 (10687.01, 20920.20)	−15.22	<0.001*
Miscellaneous service fee	1864.87 ± 803.79	1672.5 (1400.02, 2022.79)	1804.77 ± 981.62	1593.89 (1296.95, 2056.12)	−2.46	0.014*
Diagnostic fee	6375.22 ± 1780.59	6151.77 (5024.41, 7492.75)	6692.21 ± 1885.63	6625.13 (5346.91, 7759.98)	−2.79	0.005*
Treatment fee	10159.19 ± 1599.59	9,498 (9278.70, 10810.15)	10481.86 ± 2428.97	10710.2 (8341.58, 11814.60)	−1.66	0.098
Rehabilitation fee	210.57 ± 198.43	163.2 (81.60, 322.58)	245.09 ± 267.75	163.2 (61.20, 341.70)	−0.79	0.432
Western Medicine cost	2853.96 ± 1083.3	2698.83 (2195.10, 3211.87)	2036.63 ± 952.85	1865.95 (1451.57, 2444.75)	−13.99	<0.001*
Total Chinese medicine cost	872.75 ± 624.87	814.15 (500.25, 1067.29)	443.85 ± 523.95	243.7 (107.20, 605.55)	−12.58	<0.001*
Blood and Blood Products cost	157.76 ± 457.39	0 (0.00, 0.00)	136.88 ± 409.83	0 (0.00, 0.00)	−0.57	0.569
Consumables cost	42829.83 ± 7064.75	40120.09 (39079.48, 45498.63)	11137.09 ± 6339.88	9964.27 (8380.49, 12348.62)	−25.42	<0.001*

^a^The exchange rate of 100 USD equals approximately 661 CNY (1st May 2019 exchange rate).

^*^p-value<0.05.

The total expenses for TKA inpatients (*Z* = −25.15, *p* < 0.001) showed a statistically significant difference after policy implementation. The total expenses decreased from 65324.73 CNY per case to 34465.57 CNY per case, representing a reduction of 30859.16 CNY per case. The consumables cost for TKA inpatients (*Z* = −25.42, *p* < 0.001) also showed a significant difference, decreasing from 42829.83 CNY per case to 11,137.09 CNY per case, representing a reduction of 31,692.74 CNY per case. The self-financed expenses (*Z* = −15.22, *p* < 0.001), miscellaneous service fees (*Z* = −2.46, *p* < 0.05), western medicine costs (*Z* = −13.99, *p* < 0.001), and total Chinese medicine costs (*Z* = −12.58, *p* < 0.001) statistically slightly decreased after policy implementation. However, the diagnostic fees (*Z* = −2.97, *p* < 0.050) showed an increase after policy implementation.

The comparison of total expenses of TKA inpatients before and after policy implementation is shown in Table 3. Before policy implementation, the majority of TKA inpatients had total expenses in the range of 60,000 to 79,999 CNY, accounting for 193 cases (66.6%). The lowest number of cases was observed among TKA in patients with total expenses of 80,000 CNY or more, accounting for 19 cases (6.6%). After the implementation, the proportion of TKA in patients with total expenses in the range of 20,000 to 39,999 CNY increased from 0 to 88.3%. On the other hand, the proportion of TKA in patients with total expenses in the range of 40,000 to 59,999 CNY, 60,000 to 79,999 CNY, and 80,000 CNY or more all decreased.

**Table 3:** The frequency distribution of total expenses among TKA inpatients before and after policy implementation.

Total expense (CNY ^a^/n)	Before NVBP		After NVBP		Total	
	*n*	Component ratio	*n*	Component ratio	*n*	Component ratio
20,000 ~ 39,999	0	0%	800	88.3%	800	66.89%
40,000 ~ 59,999	78	26.9%	95	10.5%	173	14.46%
60,000 ~ 79,999	193	66.6%	7	0.8%	200	16.72%
>80,000	19	6.6%	4	0.4%	23	1.92%
Total	290	100%	906	100%	1,196	100%

^a^The exchange rate of 100 USD equals approximately 661 CNY (1st May 2019 exchange rate).

^*^p-value<0.05.

As shown in Table 4, we conducted a grey relation analysis to rank the influencing components of total expenses. The results revealed that the top three influencing factors on the total expense were self-financed expenses, consumables costs, and treatment fees, with correlation coefficients of 0.809, 0.788, and 0.741, respectively. On the other hand, rehabilitation fees and blood and blood product costs had the least impact on the total expense, with correlation coefficients of 0.680 each.

**Table 4:** The ranking of influencing components of total expense among TKA inpatients.

Itemized Expenses	Correlation coefficients	Rank
Self-financed expense	0.809	1
Consumables cost	0.788	2
Treatment fee	0.741	3
Diagnostic fee	0.717	4
Western medicine cost	0.691	5
Miscellaneous service fee	0.689	6
Total Chinese medicine cost	0.682	7
Rehabilitation fee	0.680	8
Blood and blood products cost	0.680	9

#### Consumables cost of TKA inpatients before and after NVBP

Basic information on the costs of consumables for TKA in patients before and after policy implementation is shown in Table 5. Before policy implementation, the majority of TKA inpatients had consumable costs in the range of 40,000 to 59,999 CNY, accounting for 168 cases (57.9%). The lowest number of cases was observed among TKA inpatients with consumables costs of 80,000 CNY or more, accounting for 1 case (0.3%). After the implementation of the policy, the proportion of TKA in patients with consumables costing less than CNY 20,000 increased from 0 to 97.6%, with a total of 884 cases. On the other hand, the proportions of TKA in patients with consumable costs in the ranges of 20,000 to 39,999 CNY, 40,000 to 59,999 CNY, 60,000 to 79,999 CNY, and 80,000 CNY or more all decreased.

**Table 5:** The frequency distribution of consumables costs among TKA inpatients before and after policy implementation.

Consumables cost (CNY ^a^/n)	Before NVBP		After NVBP		Total	
	*n*	component ratio	*n*	component ratio	*n*	component ratio
<20,000	0	0%	884	97.6%	884	73.91%
20,000 ~ 39,999	117	40.3%	18	2%	135	11.29%
40,000 ~ 59,999	168	57.9%	2	0.2%	170	14.21%
60,000 ~ 79,999	4	1.4%	1	0.1%	5	0.42%
>80,000	1	0.3%	1	0.1%	2	0.17%
Total	290		906		1,196	

^a^The exchange rate of 100 USD equals approximately 661 CNY (1st May 2019 exchange rate).

#### The interrupted time series analysis results

ITS analysis results are presented in Table 6. Upon the implementation of the procurement policy (May 1, 2022), TKA inpatients exhibited decreasing trends in total expenses ( $\beta_{2}=$ −28240.17, *p* < 0.001), consumables costs ( $\beta_{2}$ = − 31302.72, p < 0.001), and self-financed expenses ( $\beta_{2}=$ −13674.56, *p* < 0.001). Conversely, there were increases in miscellaneous service fees ( $\beta_{2}$ = 440.45, *p*<0.05), diagnostic fees ( $\beta_{2}$ =746.0, *p* < 0.05), and rehabilitation fees ( $\beta_{2}$ =207.36, *p* < 0.001), with statistically significant differences observed. The remaining indicators did not show statistically significant differences (All *p*-values≥ 0.05 ).

**Table 6:** Interrupted time series analysis for total and itemized expenses among TKA inpatients before and after policy implementation.

Variables	Constantβ_0_	Before the implementation of NVBP			When implementing NVBP		After the implementation of NVBP			
		Secular change β_1_	SE	*t*	Level change β_2_	SE	*t*	Trend change β_3_	SE	*t*
Total expense	64467.42*	29.69	42.52	0.70	−28240.17*	1627.33	−17.35	−106.95*	46.40	−2.31
Self-financed expense	30602.28*	70.21	69.12	1.02	−13674.56*	1912.58	−7.15	−137.66	71.63	−1.92
Consumables cost	41784.43*	36.03	27.38	1.32	−31302.72*	1094.64	−28.60	−65.05*	29.14	−2.23
Miscellaneous service fee	2094.89*	−9.04	6.32	−1.43	440.45*	182.16	2.42	3.42	6.68	0.51
Diagnostic fee	6163.55*	8.44	9.55	0.88	746.00*	301.19	2.48	−22.44*	10.43	−2.15
Treatment cost	9858.27*	12.20	10.53	1.16	689.17	460.24	1.50	−28.01*	11.83	−2.37
Rehabilitation fee	267.60*	−2.05	1.79	−1.14	207.36*	56.60	3.66	−0.88	1.96	−0.45
Total Chinese medicine cost	876.37*	0.26	3.45	0.08	−25.27	149.55	−0.17	−9.98*	3.79	−2.64
Western medicine cost	3367.71*	−20.41*	6.15	−3.32	486.12	251.69	1.93	4.16	6.28	0.66
Blood and blood products cost	55.90*	3.38	2.20	1.53	13.87	82.85	0.17	−5.88*	2.41	−2.44

^*^p-value<0.05.

After the policy implementation period (from May 1, 2022, to December 26, 2023), TKA inpatients showed decreasing trends in total expenses ( $\beta_{3}=-$ 106.95, *p* < 0.05), consumables costs ( $\beta_{3}=$ −65.05, *p* < 0.05), diagnostic fees ( $\beta_{3}=$ −22.44, *p* < 0.05), treatment costs ( $\beta_{3}=$ −28.01, p < 0.05), total Chinese medicine costs( $\beta_{3}=$ −9.98, *p* < 0.05), and blood and blood products fees( $\beta_{3}=$ −5.88, *p* < 0.05).

According to the component ranking (Table 4), the ITS analysis was conducted on the total and the four most relevant itemized hospitalization expenses across subgroups based on the length of hospitalization (Appendix Table 3). For TKA inpatients of any length of stay, a reduction in total expenses, self-financed expenses, and consumables costs was observed following the implementation of the NVBP on May 1, 2022 (all *p*-values<0.05). After the policy implementation period (from May 1, 2022, to December 26, 2023), treatment fees showed a slightly increasing trend. In contrast, diagnostic fees increased for only TKA inpatients with a hospital stay ≥10 days ( $\beta_{2}$ =864.25, *p* = 0.025) but exhibited a slight decreasing trend thereafter ( $\beta_{3}$ = − 35.206, *p* = 0.001).

**Figure 1:**
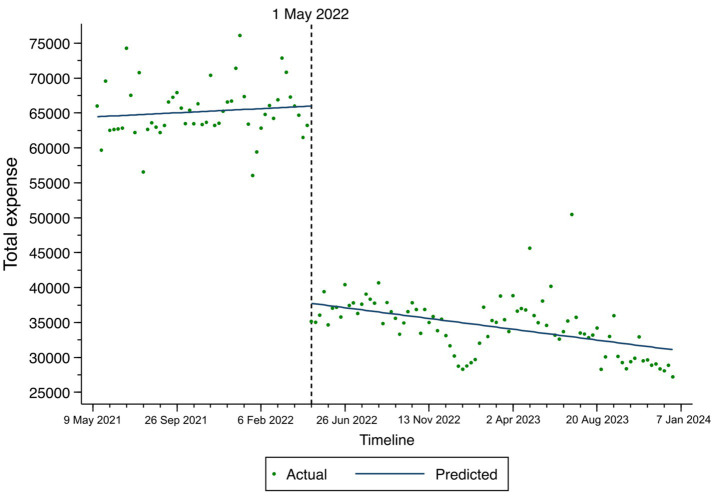
Fitted trend of total expenses changes among TKA inpatients before and after policy implementation based on the ITS model.

**Figure 2:**
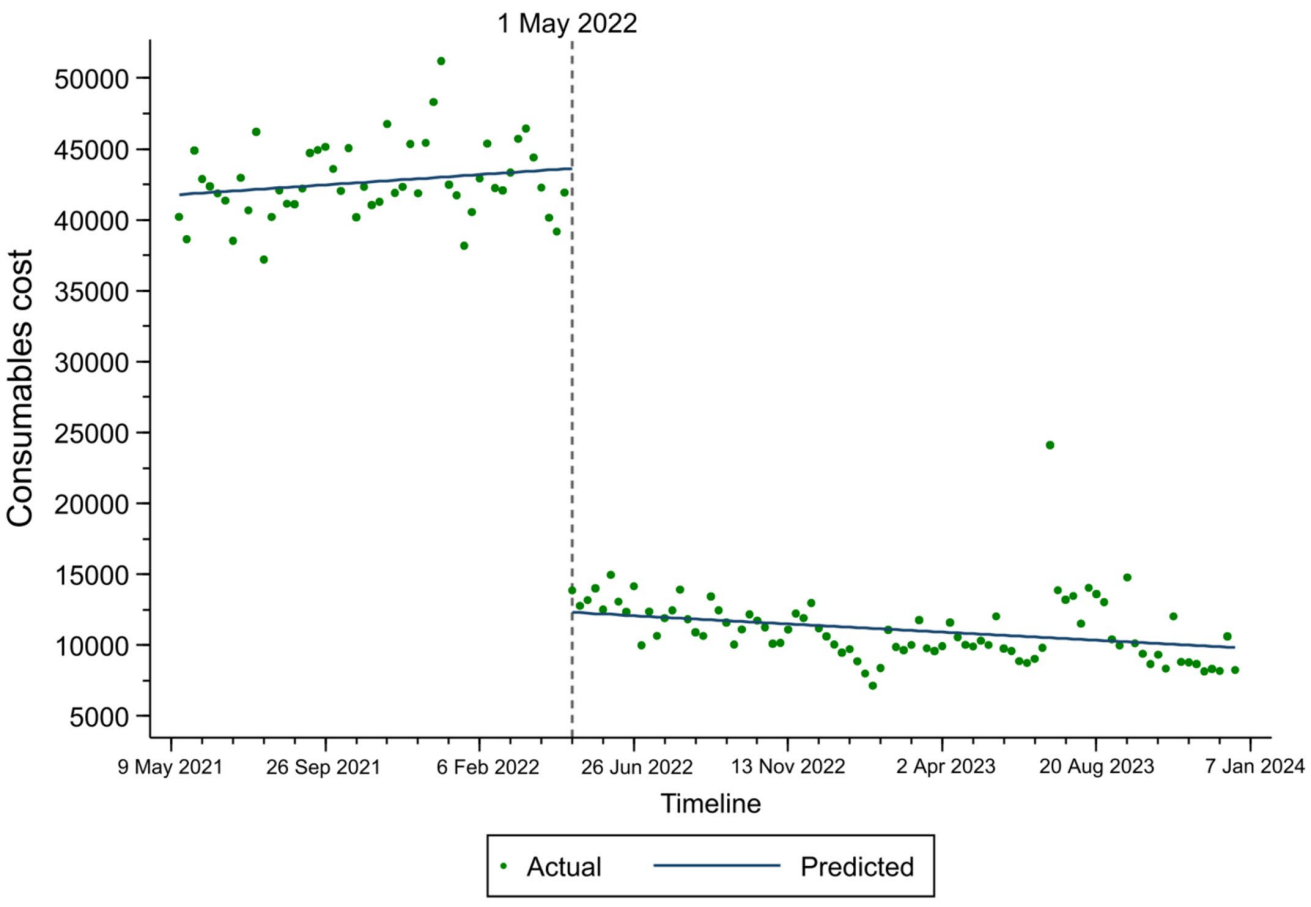
Fitted trend of consumables costs changes among TKA inpatients before and after policy implementation based on the ITS model.

**Figure 3:**
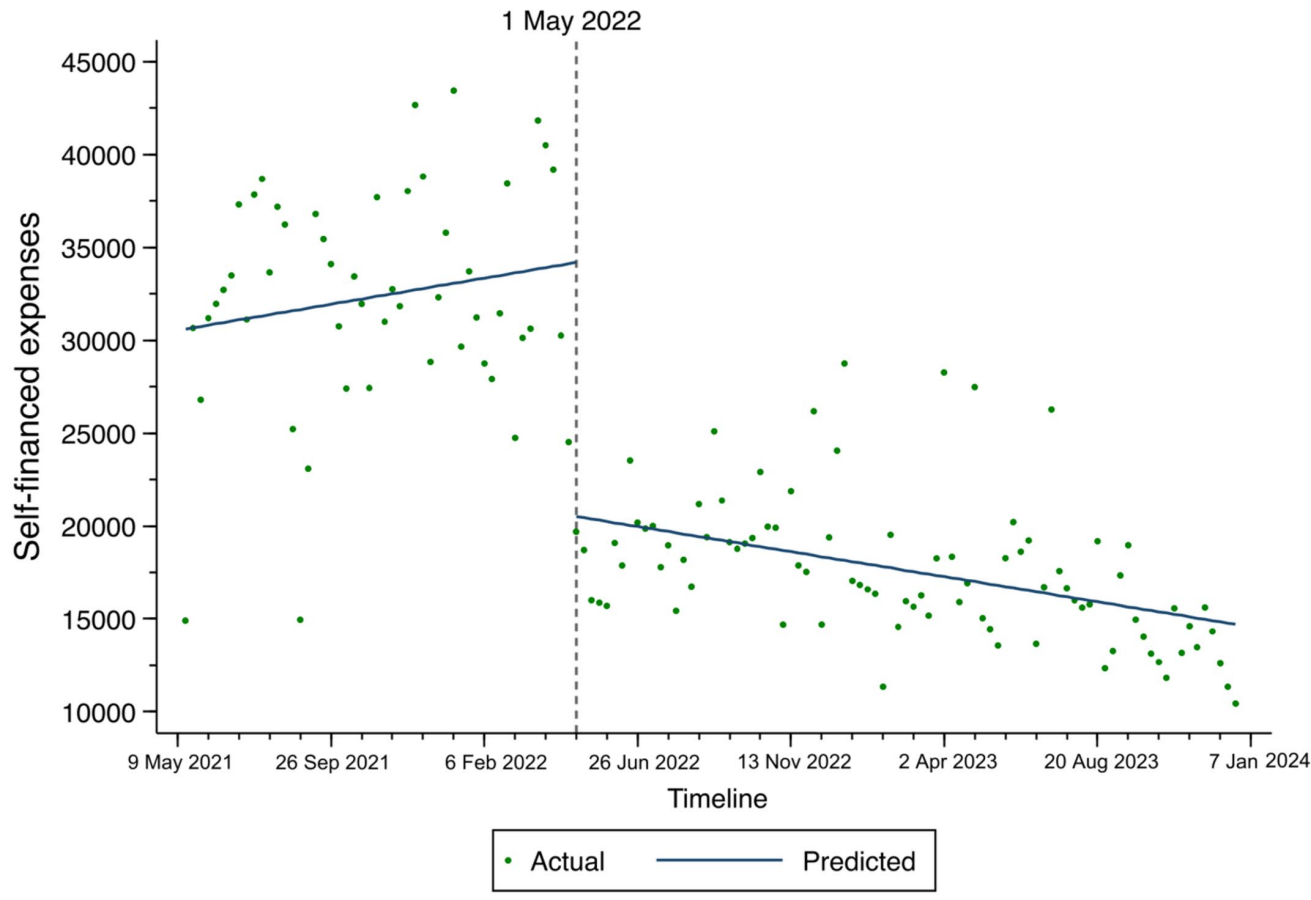
Fitted trend of self-financed expenses changes among TKA inpatients before and after policy implementation based on the ITS model.

The changes in total expenses, consumables costs, and self-financed expenses among TKA inpatients based on the ITS result are illustrated in Figures 1–3. At the time of policy implementation (May 1, 2022), the total expenses (Figure 1) exhibited a significant instantaneous drop, decreasing by 30,859.16 CNY per case. Similarly, the consumables costs (Figure 2) also showed an instantaneous drop, decreasing by 31,692.74 CNY per case. The self-financed expenses (Figure 3) displayed a slight decrement, reducing by 15,165.1 CNY per case. Further details regarding the changing trends of other variables among TKA inpatients can be found in Appendix Figures 1–7.

## Discussion

This study investigated the hospitalization expenses of patients undergoing TKA at a tertiary hospital in Guangzhou, China, from May 10, 2021, to December 26, 2023. The primary aim was to evaluate the direct and indirect impacts of the NVBP policy on the hospitalization costs of TKA patients. The empirical findings presented in this study are intended to inform policy refinement and enhance the effectiveness of patient savings. Understanding the pricing mechanisms of medical consumables under government regulation is crucial in this context.

Overall, the short-term impact of the policy was significant. Both total expenditure and the cost of consumables fell immediately after the policy was implemented, and these costs continued to fall thereafter.

First, the ITS analysis revealed that the implementation of the NVBP policy had a significant impact on total expenses, resulting in an immediate decrease of 30,859.16 CNY per case. This demonstrates the direct effect of the NVBP in controlling overall costs. In the post-implementation phase (May 1, 2022, to December 26, 2023), a consistent downward trend in total expenses was observed, further indicating a reduction and effective control over the overall costs for TKA inpatients. The grey correlation analysis identified self-financed expenses, consumable costs, and treatment fees as the most closely associated factors with total expenses. Based on these correlations, it is recommended that healthcare professionals adhere strictly to guidelines and prioritize medications listed in the national essential drug list (which are covered by medical insurance) to help regulate self-financed expenses. Additionally, the grey correlation analysis showed that miscellaneous service fees and rehabilitation fees for medical staff constituted a smaller proportion of total costs. This distribution contrasts with the allocation of treatment and nursing fees for medical personnel in Taiwan and the United States, highlighting notable differences in cost structures (27).

Second, the consumables cost experienced an immediate decrease at the time of policy implementation (May 1, 2022), with a reduction of 31,692.74 CNY. A downward trend in consumables cost was also observed after the policy implementation (from May 1, 2022, to December 26, 2023). The finding underscores the considerable influence of the NVBP program on artificial joints, aligning with current studies suggesting that NVBP can substantially reduce medication expenses and enhance medication benefits for patients (28–30). Previously, the pricing of high-value medical consumables in China was mainly determined by companies. It often resulted in inflated prices due to a lack of transparency in the market and non-standardized operations. Now, the ITS analysis in this study showed a significant decrease in consumables costs under the policy’s influence. Additionally, the correlation analysis revealed that the second most influential factor on TKA inpatients’ total expense was average consumables cost. The NVBP policy undoubtedly reduced consumables costs, thereby alleviating the overall burden on TKA inpatients. China can also learn from the experiences of other countries. Nevertheless, the overall performance of orthopedic surgeons may be influenced by a reduction in surgical costs. The issue of subjective motivation among orthopedic surgeons requires further investigation. In the United States, the implementation of surgeon scorecards has enabled cost comparisons among peers, effectively motivating surgeons to participate in reducing the cost of TKA (31).

Third, the ITS analysis also revealed a decrease in self-financed expenses following the policy implementation. According to the National Bureau of Statistics of China, the per capita disposable income of Chinese residents was approximately 28,200 CNY in 2018 (27). Under the NVBP, the mediation of self-financed expenses was reduced to 16,864.9 CNY, making it more affordable for patients. This reduction in out-of-pocket costs significantly alleviates the financial burden on patients. Previous studies from various countries, including China, have indicated that TKA is a highly cost-effective procedure for patients (15, 32, 33). Our findings further demonstrate that the NVBP leads to greater reductions in TKA-related expenses, thereby increasing the opportunities for arthritis patients to enhance their quality of life.

In addition, when the implementation of the policy (May 1, 2022) occurred, miscellaneous service fees, diagnostic fees, and rehabilitation fees showed an immediate but slight increase. In China, a reflection of the value of technical labor includes miscellaneous service fees and rehabilitation fees. This study observed an immediate increase in these two variables before and after the policy implementation. This may suggest that the value of medical professionals’ work was being better recognized. The structure of total expenses became more reasonable, in line with China’s policy direction for the quality development of public hospitals (34). However, as a non-technical labor cost, the diagnostic fee also exhibited an immediate upward trend in this study. The grey correlation analysis showed a close relationship between the diagnostic fee and total expense. Research on the centralized procurement of intraocular lenses also showed that the diagnostic fee accounted for a larger proportion of patients’ hospitalization costs (35). In this study, diagnostic fees were broken down into several components, including pathological diagnosis, laboratory testing, and medical imaging. Diagnostic costs increased for inpatients with longer hospital stays, who usually need more extensive screenings and diagnostic evaluations during prolonged hospitalization, more likely due to affordability after NVBP and flexibility of osteoarthritis patients. As overall hospitalization costs decrease and treatments become more affordable due to policy changes, orthopedic surgeons gain more flexibility to develop comprehensive and careful treatment plans for osteoarthritis patients. This shift allows for a more patient-centered approach to care. However, it is equally important to emphasize careful and individualized treatment plans for patients with shorter hospital stays, where no significant expense changes were observed in the study. For these patients, timely interventions and accurate assessments are crucial to ensure effective treatment and prevent possible premature discharge or inadequate care planning from hindering recovery. By striking a balance between cost-effective management and personalized care, the healthcare system can improve both efficiency and patient outcomes.

However, after the policy implementation (from May 1, 2022, to December 26, 2023), there was a slight downward trend in diagnostic fees, treatment fees, total Chinese medicine costs, and blood and blood products costs for TKA inpatients. Previous research has shown that medication costs and consumable costs, as two major components of hospital revenue, seem to have a compensatory relationship. Other policies in China restricting medication profitability produced comparable results: a reduction in the proportion of drug costs was accompanied by an increase in consumable expenses (19). In this study, the decrease in consumable costs was associated with a downward trend in total Chinese medicine costs. It was understood that the hospital management made adjustments to the charging items to optimize the fee structure, which might be a reason for the reduction in various costs. Therefore, when formulating the volume-based procurement policy for artificial consumables, government departments should take into account the adjustment of other cost structures and provide clear cost standards for other items. In practical terms, NVBP faces the challenge that the interests of healthcare companies cannot be guaranteed (36). A compensation mechanism for healthcare institutions should be explored and implemented.

Based on the findings, the volume-based procurement policy for artificial joints requires further measures to protect and recognize the interests and values of healthcare providers. China’s national procurement guidelines emphasize the importance of supporting measures, primarily focusing on increasing the proportion of medical service revenue and optimizing compensation systems to reflect healthcare professionals’ labor value (37). However, hospital administrators in this study reported that the overall reduction in surgical costs has notably impacted departmental performance metrics. Enhancing healthcare providers’ motivation to participate in high-value medical consumable reforms remains an area requiring policy refinement and improvement.

## Limitation

This study has several limitations. First, it is a single-center study conducted at a tertiary hospital in Guangzhou, which may limit the generalizability of the findings to a broader population. The results may not fully reflect the nationwide implementation of volume-based procurement of artificial joints in China, and regional differences in economic conditions and healthcare needs should be considered when interpreting the findings. Second, while a single-group ITS analysis allows for the inclusion of time-related factors, certain confounding variables may not have been fully accounted for. The study only conducted limited stratified analyses of hospitalization costs. Future research using a multi-group ITS analysis and a broader dataset could provide a more comprehensive understanding of the intervention’s overall effects and variations across different patient groups. A more detailed examination of how individual characteristics influence outcomes would also be beneficial. Third, this study focused primarily on the affordability of hospitalization costs for patients. However, a more thorough exploration of health outcomes and the quality of medical services provided to TKA patients is necessary to fully assess the policy’s impact on patient well-being. Future studies should aim to examine the broader implications of the policy on healthcare quality, thereby providing a more holistic evaluation of its effects. In summary, future research should seek to improve generalizability across different regions, account for confounding factors, and investigate the wider effects on health outcomes and the quality of care to offer a more comprehensive assessment of the policy’s impact.

## Conclusion

Given China’s large and aging population, the demand for total knee arthroplasty surgery is steadily increasing. This study examines both the direct and indirect impacts of the NVBP policy on the costs associated with TKA inpatient care, offering empirical evidence to inform government policy optimization. The findings suggest that the NVBP for artificial joints in Guangdong Province has largely met its intended goals. The government plays a crucial role in shaping the pricing and distribution of medical consumables. The study reveals significant reductions in total expenses, consumable costs, and self-financed expenses for TKA patients, which enhances the accessibility of healthcare services and supports patient recovery. Moreover, while labor-related costs for healthcare professionals have seen a modest increase, the quality of treatment and medical planning has also improved. Ongoing attention to fluctuations in various fees is necessary. To further enhance the efficacy of the volume-based procurement policy, the government should consider adjusting medical service prices and refining compensation mechanisms to better motivate healthcare providers.

## Data Availability

The raw data supporting the conclusions of this article will be made available by the authors, without undue reservation.
