# Supplementary material for: A comparative study of hospitalization costs of TKA inpatients before and after National Volume-based Procurement in Guangdong, China: an interrupted time-series analysis
Source: Front Public Health. 2025 Jan 9;12:1468606. doi: 10.3389/fpubh.2024.1468606 (PMC11754277; doi:10.3389/fpubh.2024.1468606)
Supplement: Supplementary file 1 [file Supplementary_file_1.docx]

Appendix Table 1 Assignment of segmented linear regression model

| **point in time**  **(year, week)** | **Before the implementation of NVBP** | | | | **point in time**  **(year, week)** | **When implementing NVBP** | | | | **point in time**  **(year, week)** | **After the implementation of NVBP** | | | |
| --- | --- | --- | --- | --- | --- | --- | --- | --- | --- | --- | --- | --- | --- | --- |
|  | **dependent variable** | ***X1*** | ***X*2** | ***X*3** |  | **dependent variable** | ***X1*** | ***X*2** | ***X*3** |  | **dependent variable** | ***X1*** | ***X*2** | ***X*3** |
| 2021,21 | *Y*1 | 1 | 0 | 0 | 2022,20 | *Y*53 | 53 | 1 | 0 | 2023,19 | *Y*105 | 105 | 1 | 52 |
| 2021,22 | *Y*2 | 2 | 0 | 0 | 2022,21 | *Y*54 | 54 | 1 | 1 | 2023,20 | *Y*106 | 106 | 1 | 53 |
| 2021,23 | *Y*3 | 3 | 0 | 0 | 2022,22 | *Y*55 | 55 | 1 | 2 | 2023,21 | *Y*107 | 107 | 1 | 54 |
| 2021,24 | *Y*4 | 4 | 0 | 0 | 2022,23 | *Y*56 | 56 | 1 | 3 | 2023,22 | *Y*108 | 108 | 1 | 55 |
| 2021,25 | *Y*5 | 5 | 0 | 0 | 2022,24 | *Y*57 | 57 | 1 | 4 | 2023,23 | *Y*109 | 109 | 1 | 56 |
| 2021,26 | *Y*6 | 6 | 0 | 0 | 2022,25 | *Y*58 | 58 | 1 | 5 | 2023,24 | *Y*110 | 110 | 1 | 57 |
| 2021,27 | *Y*7 | 7 | 0 | 0 | 2022,26 | *Y*59 | 59 | 1 | 6 | 2023,25 | *Y*111 | 111 | 1 | 58 |
| 2021,28 | *Y*8 | 8 | 0 | 0 | 2022,27 | *Y*60 | 60 | 1 | 7 | 2023,26 | *Y*112 | 112 | 1 | 59 |
| 2021,29 | *Y*9 | 9 | 0 | 0 | 2022,28 | *Y*61 | 61 | 1 | 8 | 2023,27 | *Y*113 | 113 | 1 | 60 |
| 2021,30 | *Y*10 | 10 | 0 | 0 | 2022,29 | *Y*62 | 62 | 1 | 9 | 2023,28 | *Y*114 | 114 | 1 | 61 |
| 2021,31 | *Y*11 | 11 | 0 | 0 | 2022,30 | *Y*63 | 63 | 1 | 10 | 2023,29 | *Y*115 | 115 | 1 | 62 |
| 2021,32 | *Y*12 | 12 | 0 | 0 | 2022,31 | *Y*64 | 64 | 1 | 11 | 2023,30 | *Y*116 | 116 | 1 | 63 |
| 2021,33 | *Y*13 | 13 | 0 | 0 | 2022,32 | *Y*65 | 65 | 1 | 12 | 2023,31 | *Y*117 | 117 | 1 | 64 |
| 2021,34 | *Y*14 | 14 | 0 | 0 | 2022,33 | *Y*66 | 66 | 1 | 13 | 2023,32 | *Y*118 | 118 | 1 | 65 |
| 2021,35 | *Y*15 | 15 | 0 | 0 | 2022,34 | *Y*67 | 67 | 1 | 14 | 2023,33 | *Y*119 | 119 | 1 | 66 |
| 2021,36 | *Y*16 | 16 | 0 | 0 | 2022,35 | *Y*68 | 68 | 1 | 15 | 2023,34 | *Y*120 | 120 | 1 | 67 |
| 2021,37 | *Y*17 | 17 | 0 | 0 | 2022,36 | *Y*69 | 69 | 1 | 16 | 2023,35 | *Y*121 | 121 | 1 | 68 |
| 2021,38 | *Y*18 | 18 | 0 | 0 | 2022,37 | *Y*70 | 70 | 1 | 17 | 2023,36 | *Y*122 | 122 | 1 | 69 |
| 2021,39 | *Y*19 | 19 | 0 | 0 | 2022,38 | *Y*71 | 71 | 1 | 18 | 2023,37 | *Y*123 | 123 | 1 | 70 |
| 2021,40 | *Y*20 | 20 | 0 | 0 | 2022,39 | *Y*72 | 72 | 1 | 19 | 2023,38 | *Y*124 | 124 | 1 | 71 |
| 2021,41 | *Y*21 | 21 | 0 | 0 | 2022,40 | *Y*73 | 73 | 1 | 20 | 2023,39 | *Y*125 | 125 | 1 | 72 |
| 2021,42 | *Y*22 | 22 | 0 | 0 | 2022,41 | *Y*74 | 74 | 1 | 21 | 2023,40 | *Y*126 | 126 | 1 | 73 |
| 2021,43 | *Y*23 | 23 | 0 | 0 | 2022,42 | *Y*75 | 75 | 1 | 22 | 2023,41 | *Y*127 | 127 | 1 | 74 |
| 2021,44 | *Y*24 | 24 | 0 | 0 | 2022,43 | *Y*76 | 76 | 1 | 23 | 2023,42 | *Y*128 | 128 | 1 | 75 |
| 2021,45 | *Y*25 | 25 | 0 | 0 | 2022,44 | *Y*77 | 77 | 1 | 24 | 2023,43 | *Y*129 | 129 | 1 | 76 |
| 2021,46 | *Y*26 | 26 | 0 | 0 | 2022,45 | *Y*78 | 78 | 1 | 25 | 2023,44 | *Y*130 | 130 | 1 | 77 |
| 2021,47 | *Y*27 | 27 | 0 | 0 | 2022,46 | *Y*79 | 79 | 1 | 26 | 2023,45 | *Y*131 | 131 | 1 | 78 |
| 2021,48 | *Y*28 | 28 | 0 | 0 | 2022,47 | *Y*80 | 80 | 1 | 27 | 2023,46 | *Y*132 | 132 | 1 | 79 |
| 2021,49 | *Y*29 | 29 | 0 | 0 | 2022,48 | *Y*81 | 81 | 1 | 28 | 2023,47 | *Y*133 | 133 | 1 | 80 |
| 2021,50 | *Y*30 | 30 | 0 | 0 | 2022,49 | *Y*82 | 82 | 1 | 29 | 2023,48 | *Y*134 | 134 | 1 | 81 |
| 2021,51 | *Y*31 | 31 | 0 | 0 | 2022,50 | *Y*83 | 83 | 1 | 30 | 2023,49 | *Y*135 | 135 | 1 | 82 |
| 2021,52 | *Y*32 | 32 | 0 | 0 | 2022,51 | *Y*84 | 84 | 1 | 31 | 2023,50 | *Y*136 | 136 | 1 | 83 |
| 2021,53 | *Y*33 | 33 | 0 | 0 | 2022,52 | *Y*85 | 85 | 1 | 32 | 2023,51 | *Y*137 | 137 | 1 | 84 |
| 2022,01 | *Y*34 | 34 | 0 | 0 | 2022,53 | *Y*86 | 86 | 1 | 33 | 2023,52 | *Y*138 | 138 | 1 | 85 |
| 2022,02 | *Y*35 | 35 | 0 | 0 | 2023,01 | *Y*87 | 87 | 1 | 34 |  |  |  |  |  |
| 2022,03 | *Y*36 | 36 | 0 | 0 | 2023,02 | *Y*88 | 88 | 1 | 35 |  |  |  |  |  |
| 2022,04 | *Y*37 | 37 | 0 | 0 | 2023,03 | *Y*89 | 89 | 1 | 36 |  |  |  |  |  |
| 2022,05 | *Y*38 | 38 | 0 | 0 | 2023,04 | *Y*90 | 90 | 1 | 37 |  |  |  |  |  |
| 2022,06 | *Y*39 | 39 | 0 | 0 | 2023,05 | *Y*91 | 91 | 1 | 38 |  |  |  |  |  |
| 2022,07 | *Y*40 | 40 | 0 | 0 | 2023,06 | *Y*92 | 92 | 1 | 39 |  |  |  |  |  |
| 2022,08 | *Y*41 | 41 | 0 | 0 | 2023,07 | *Y*93 | 93 | 1 | 40 |  |  |  |  |  |
| 2022,-9 | *Y*42 | 42 | 0 | 0 | 2023,08 | *Y*94 | 94 | 1 | 41 |  |  |  |  |  |
| 2022,10 | *Y*43 | 43 | 0 | 0 | 2023,09 | *Y*95 | 95 | 1 | 42 |  |  |  |  |  |
| 2022,11 | *Y*44 | 44 | 0 | 0 | 2023,10 | *Y*96 | 96 | 1 | 43 |  |  |  |  |  |
| 2022,12 | *Y*45 | 45 | 0 | 0 | 2023,11 | *Y*97 | 97 | 1 | 44 |  |  |  |  |  |
| 2022,13 | *Y*46 | 46 | 0 | 0 | 2023,12 | *Y*98 | 98 | 1 | 45 |  |  |  |  |  |
| 2022,14 | *Y*47 | 47 | 0 | 0 | 2023,13 | *Y*99 | 99 | 1 | 46 |  |  |  |  |  |
| 2022,15 | *Y*48 | 48 | 0 | 0 | 2023,14 | *Y*100 | 100 | 1 | 47 |  |  |  |  |  |
| 2022,16 | *Y*49 | 49 | 0 | 0 | 2023,15 | *Y*101 | 101 | 1 | 48 |  |  |  |  |  |
| 2022,17 | *Y*50 | 50 | 0 | 0 | 2023,16 | *Y*102 | 102 | 1 | 49 |  |  |  |  |  |
| 2022,18 | *Y*51 | 51 | 0 | 0 | 2023,17 | *Y*103 | 103 | 1 | 50 |  |  |  |  |  |
| 2022,19 | *Y*52 | 52 | 0 | 0 | 2023,18 | *Y*104 | 104 | 1 | 51 |  |  |  |  |  |

Appendix Table 2 Factors influencing days of hospitalization for TKA in patients (n=1196).

| **Variables** | | **β(SE)** | **t** | **95%CI** | **P** |
| --- | --- | --- | --- | --- | --- |
| **Medical insurance** (reference: Employee basic medical insurance) |  |  |  |  |  |
|  | Resident basic medical insurance | -1.06(0.511) | -2.076 | (-2.062,-0.058) | 0.038 |
|  | Fully self-pay | 1.504（0.586） | 2.568 | (0.355,2.653) | 0.010 |

Note: The table only includes significant variables.

Appendix Table 3 Interrupted time series analysis of total and four most relevant itemized hospitalization expenses for TKA inpatients with varying days of hospitalization

| **TKA inpatients** | | **Variables** | **Constant** β_0_ | **Before the implementation of NVBP** | | | | **When implementing NVBP** | | | | | **After the implementation of NVBP** | | | |
| --- | --- | --- | --- | --- | --- | --- | --- | --- | --- | --- | --- | --- | --- | --- | --- | --- |
|  |  |  |  | **Secular change** β_1_ | **SE** | **t** | **P** | | **Level change** β_2_ | **SE** | **t** | **P** | **Trend change** β_3_ | **SE** | **t** | **P** |
| Days of hospitalization | <10 days | Total expense | 64584.720* | -105.279 | 67.580 | -1.560 | 0.122 | | -19854.480 | 2460.601 | -8.070 | ＜0.001* | -19.683 | 70.372 | -0.280 | 0.780 |
|  |  | Self-financed expense | 32615.450* | -63.306 | 63.490 | -1.000 | 0.321 | | -7269.333 | 2526.362 | -2.880 | 0.006* | -52.308 | 69.421 | -0.750 | 0.452 |
|  |  | Consumables cost | 44474.210* | -118.191 | 70.331 | -1.680 | 0.095 | | -20995.600 | 2587.837 | -8.110 | ＜0.001* | 8.056 | 72.263 | 0.110 | 0.911 |
|  |  | Treatment fee | 9564.823* | 7.950 | 6.199 | 1.280 | 0.202 | | 189.767 | 264.887 | 0.720 | 0.475 | -20.059 | 6.828 | -2.940 | 0.004* |
|  |  | Diagnostic fee | 5679.184* | 8.513 | 8.272 | 1.030 | 0.305 | | 22.562 | 283.352 | 0.080 | 0.937 | -10.538 | 8.808 | -1.200 | 0.234 |
|  | ≥10 days | Total expense | 66591.070* | -74.511 | 85.802 | -0.870 | 0.387 | | -22525.310 | 3349.498 | -6.720 | ＜0.001* | -6.478 | 91.070 | -0.070 | 0.943 |
|  |  | Self-financed expense | 31215.520* | 12.210 | 71.116 | 0.170 | 0.864 | | -11155.090 | 2522.716 | -4.420 | ＜0.001* | -54.879 | 75.133 | -0.730 | 0.466 |
|  |  | Consumables cost | 43013.620* | -109.351 | 74.668 | -1.460 | 0.145 | | -24710.000 | 2825.433 | -8.750 | ＜0.001* | 82.359 | 75.721 | 1.090 | 0.279 |
|  |  | Treatment fee | 10049.150* | 25.313 | 9.157 | 2.760 | 0.007* | | 228.646 | 494.107 | 0.460 | 0.644 | -35.206 | 11.353 | -3.100 | 0.002* |
|  |  | Diagnostic fee | 6381.781* | 14.092 | 8.193 | 1.720 | 0.088 | | 864.250 | 381.613 | 2.260 | 0.025* | -32.885 | 9.476 | -3.470 | 0.001* |

* p-value＜0.05
